# An Island Flap Technique for Laryngeal Intracordal Mucous Retention Cysts 

**Published:** 2015-09

**Authors:** Farzad Izadi, Hadi Ghanbari, Sahar Zahedi, Behzad Pousti, Mojtaba Maleki Delarestaghi, Abolfazl Salehi

**Affiliations:** 1 *Department of Otorhinolaryngology, Rasoul Akram Hospital, Iran University of Medical Sciences, Tehran, Iran.*; 2 *Department of Speech Therapy, University of Social Welfare and Rehabilitation Sciences, Tehran, Iran.*

**Keywords:** CO2 laser, Island flap, Mucous retention cyst, Voice analysis.

## Abstract

**Introduction:**

Mucous retention cysts are a subtype of intracordal vocal cysts that may occur spontaneously or may be associated with poor vocal hygiene, and which require optimal treatment. The objective of this study was to present a new laser-assisted microsurgery technique for treating intracordal mucous retention cysts and to describe the final outcomes.

**Materials and Methods:**

In this prospective study, we assessed the pre-operative and post-operative acoustic analysis, maximum phonation time (MPT), and voice handicap index (VHI) of four patients with a diagnosis of mucous retention cyst. The island flap technique was applied to all patients without any complications. In this procedure, we favored the super-pulse mode using a 2-W power CO2 laser to remove the medial wall of the cyst, before clearing away the lateral wall margins of the cyst using repeat-pulse mode and a 2-W power CO2 laser. Indeed, we maintained the underlying epithelium and lamina propria, including the island flap attached to the vocal ligament.

**Results:**

There was a statistically significant improvement in the MPT (pre-op,11.05 s; post-op,15.85 s; P=0.002) and the VHI (pre-operative, 72/120; post-operative,27/120; P=0.001) in all patients. Moreover, jitter and shimmer were refined after surgery, but there was no statistically significant relationship between pre-operative and post-operative data (P=0.071) (P=0.622). In the follow-up period (median, 150 days), there was no report of recurrence or mucosal stiffness.

**Conclusion:**

The island flap procedure in association with CO2 laser microsurgery appears to be a safe and effective treatment option for intracordal mucous retention cysts, but needs further investigation to allow comparison with other methods.

## Introduction

Intracordal cysts are among the most common causes of dysphonia, notably in individuals who rely on their voice professionally (1). These benign lesions require challenging treatment methods and a prolonged recovery period compared with either nodules or polyps (2). Intracordal cysts are most typical in mid-membranous vocal folds and derive from the superficial layer of the lamina propria but can extend to embrace the middle and, rarely, the deep layers of the lamina propria (3,4). 

Management of vocal fold cysts provides an insight into the development of voice surgery (5). The optimal surgical technique, leading to minimum risk of recurrence and mucosal stiffness, is to preserve the maximum superficial layer of the lamina propria (6). Vocal fold cysts traditionally fall in two categories: epidermoid/keratin cysts and mucous retention cysts. Due to ductal obstruction, mucous retention cysts covered by ciliated cylindrical epithelium manifest, and often originate, just below the free margin of the fold, with significant medial projection. Epidermoid cysts possess a pearl-like aspect and project from the fold to a lesser extent. Voice abuse and residue of the epithelium trapped inside the lamina propria may cause this type of cyst (7,8). Cysts within the vocal fold lamina propria may lead to the greatest adverse effect on the vibratory characteristics of the non-neoplastic lesions. The mucosal wave is persistently absent, and aperiodic if present (9). 

For treatment of mucous retention cysts, some authors advocate phonosurgery using cold instruments, such as marsupialization or wide opening of the cysts (10). Matar et al. defined the Acublade CO_2_ laser system as a reliable treatment option for vocal cysts, with minimal mechanical and thermal damage to the epithelium, lamina propria, and vocal ligament (11). The purpose of the current study was to describe the island flap technique using CO_2_ laser-assisted microsurgery in the treatment of mucous retention cysts, and to compare pre- and post-operative voice analysis results.

## Materials and Methods

Patients undergoing CO2 laser-assisted surgery for a mucous retention cyst larger than 2×2 mm between 2011 and 2012 were included, with approval of the medical ethics committee. A detailed medical history and clinical examination of the head and neck were performed. The analysis of parameters included gender, age, voice abuse, gastroesophageal reflux symptoms, associated laryngeal lesions, videostro- boscopy examinations, and surgical and histological descriptions. We measured acoustic parameters (jitter and shimmer, frequency and intensity range) and maximum phonation time (MPT) using Speech Studio Software (Laryngograph, UK) before and 1 month after surgery. Moreover, all patients completed the VHI questionnaire before and 1 month after treatment. 

**Fig 1:**
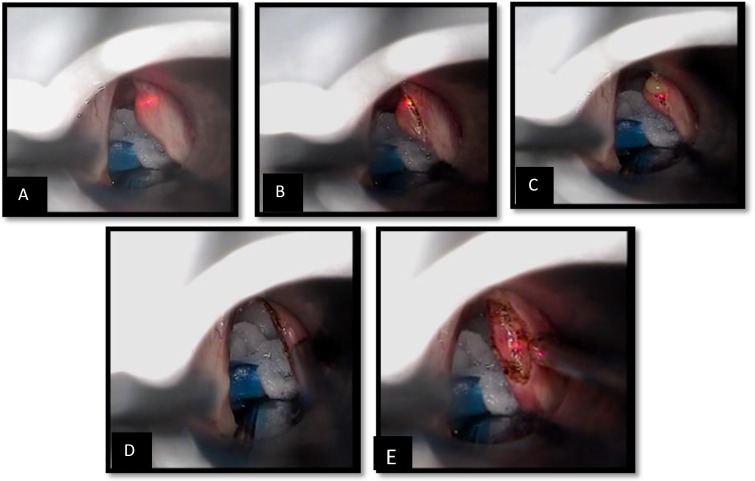
The island flap procedure. (A) Start to remove the medial wall of the cyst with CO2 laser, super pulse mode and 2 watt power. (B) and (C) Incision of the medial wall of the cyst (D). After removal of the medial wall and start to clear away the margins of the lateral wall (E) Island Flap, the preserved intact epithelium and mucous attached to the vocal ligament

**Fig 2:**
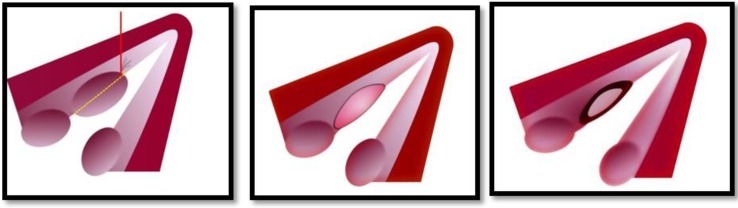
Shematic drawing of Island Flap procedure in three steps

Following general anesthesia and oral intubation using the smallest size of tracheal tube, the larynx was exposed completely with the laryngoscope (Storz, Germany). After accurate visualization of the glottis, under high magnification using an operative microscope with a 400-mm objective lens (Leica, Germany), the medial wall of the cyst was resected with super-pulse mode and a 2-watt power CO2 laser (Coherent, USA). In the second step, a repeated mode, 2-watt CO2 laser was used to spot the margins of the lateral wall in order to minimize any remnants of the secretary epithelium and thus, recurrence of the cyst. Finally, we preserved an intact epithelium and lamina propria, such as the island flap attached to the vocal ligament, so that no need to dissect the capsule of the cyst from the epithelium (Figs1,2). All samples were sent for histopathologic evaluation and adequate hemostasis was obtained using saline and 1/10000 epinephrine-soaked cottonoid. All procedures lasted between 15–30 minutes, and the patients were discharged after surgery with antibiotics (cephalexin 500 mg q.i.d for 3–5 days) and a proton pump inhibitor (omeprazole 20 mg twice a day for 1 month). The first and second visits were 2 and 4 weeks after the operation, retrospectively; videostroboscopy and voice analysis were used as a means of data collection. Patients had voice therapy for at least 1 month and filled the voice handicap index (VHI) questionnaire at the end of the first month. The Wilcoxon test was used to perform statistical analysis, and statistical significance was set at P<0.05.

## Results

Five patients (three male and two female) ranging in age from 20 to 60 years and with a diagnosis of mucous retention cyst underwent the surgery between April 2011 and April 2012. One patient did not return for post-operative tests and is therefore excluded from the current study. Finally, therefore, four patients (one female and three male) were included in our prospective study. One patient had a history of previous surgery in another medical center several months ago, with recurrence of intracordal mucous retention cyst. This patient had a nodule on the opposite side of the vocal fold, and the nodule removed using a CO2 laser at the same time. 

**Fig 3:**
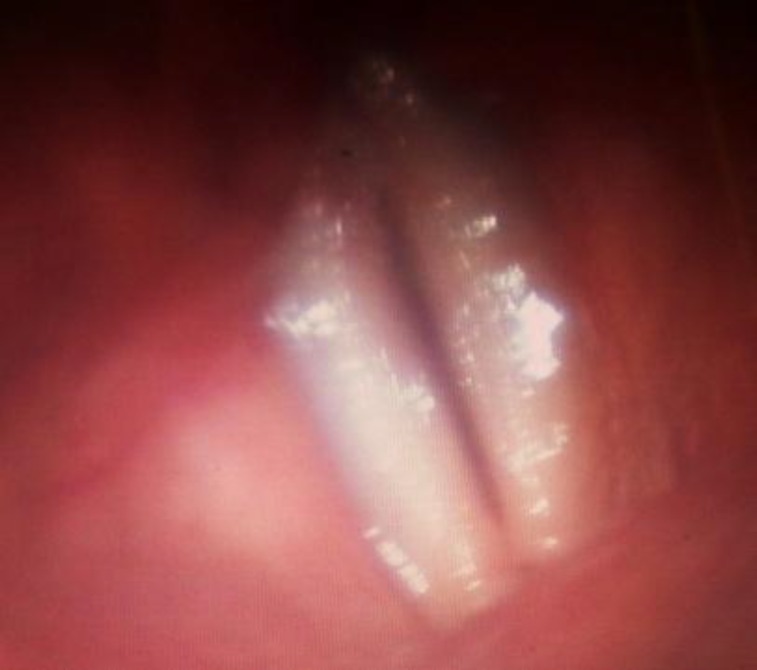
The video stroboscopic image of the same patient’s one month after surgery

Our results suggest that the MPT significantly improved from a mean value of 11.05 s before surgery to 15.85 s after surgery (P=0.002). The VHI was significantly improved from 75/120 before surgery to 27/120 after surgery (P=0.001). The jitter improved from 0.38% pre-operatively to 0.28% post-operatively (P=0.071) and the shimmer displayed improvement from 5.14% pre-operatively to 4.51% post-operatively (P=0.622); although the difference did not reach statistical significance in either case (Tables.1,2). The median period of follow-up was 11 months (range, 7–15 months), and there was no sign of recurrence or mucosal stiffness. All patients noted subjectively that their voice was improved, while post-operative stroboscopy manifested a better glottal closure and improved mucosal wave propagation in the return of incomplete glottal closure and decreased magnitude and amplitude of the mucosal wave, pre-operatively (Fig.3).

**Table1:** Pre and post-operative acoustic and aerodynamic values in 4 patients

**MPT** **Pre-op** **(ms)**	**MPT** **Post-op** **(ms)**	**Jitter** **Pre-op**	**Jitter** **Post-op**	**Shimmer** **Pre-op**	**Shimmer** **Post-op**	**VHI** **Pre-op**	**VH** **Post-op**
9	13	0.175%	0.142%	0.7%	0.580	76.120	28.120
23	29	0.701%	0.599%	6.83%	4.68	75.120	30.120
5.2	10	0.379%	0.163%	6.019%	8.53	85.120	26.120
7	11.4	0.274%	0.196%	6.839%	4.254	81.120	25.120

**Table 2:** Pre and post-operative acoustic, aerodynamic and VHI mean values

**Mean values**	**VHI**	**Jitter**	**Shimmer**	**MPT**
Pre-operative	79/120	0.38%	5.14%	11.5
Post- operative	27/120	0.28%	4.51%	15.85
P value	0.001	0.071	0.0622	0.002

## Discussion

Vocal cysts are often symptomatic and usually do not respond to medical or voice therapy (12). Surgery is the treatment of choice, and optimal surgical approaches rely upon minimal manipulation of the adjacent tissues to avoid fibrotic scars (13,14). Tai et al. suggested that the marsupialization of the cyst or use of the wide-opening method with the advantages of simplicity, minimal tissue injury, rapid functional recovery and low recurrence, can be regarded as the standard treatment of choice for medium or large vocal fold retention cysts (10). Burns and Zeitels do not advocate the use of CO2 laser as a primary choice treatment for benign superficial lesions other than malignant lesions. 

Furthermore, in their 2009 trial, they suggested that in phonosurgery of intracordal cysts, standard microflap subepithelial dissection combined with a subepithelial infusion technique can facilitate maximum preservation of pliable phonatory mucosa (superficial lamina propria and epithelium), which is deemed critical for optimal post-operative voice function (15,16). In a recent study, Martins et al. used the lateral microflap procedure as the preferred technique for the treatment of 46 patients with intracordal cysts, with satisfactory results (1). Since the inception of surgical lasers, they have increasingly been used in procedures relating to the larynx. 

In the late 1980s and early 1990s, the safety of lasers for superficial lesions of the larynx was brought into debate. Benninger suggested that the CO2 laser could be used safely on the free margin of the vocal folds. He also speculated that the major factor that has resulted in improved outcomes in the CO2 laser surgical management of vocal fold lesions has been the attention to the microanatomy and the physiological implications of laser heat distribution to the deeper layers of the lamina propria and vocalis muscle fold (17). In a recent publication, Matar et al. demonstrated the successful use of the Acublade CO2 laser system in the treatment of 49 intracordal cysts, and showed significant improvement in stroboscopic, subjective and objective evaluation at the 1-month follow-up visit. Furthermore, they injected collagen (cymetra) in the deep layers of the lamina propria in 16% of patients in order to decrease the glottal gap after resection of the cyst (11). 

In our study, we proposed a new technique for the treatment of mucous retention cysts. In this procedure, we used a CO2 laser, super-pulse mode and a 2-W power mode to resect the medial wall of the cysts. In the second step, the repeat-pulse mode and a 2-W power laser was utilized to remove the remnants of the lateral wall of the cysts. We did not dissect the cyst capsule from the underlying epithelium, and we maintained the intact epithelium and lamina propria of the vocal fold.

 The island flap procedure was used for mucous retention cysts larger than 2×2 mm and subjective and objective voice parameters were satisfactory in all patients. There was a statistically significant improvement in MPT and VHI, and both the jitter and shimmer recovered following surgery (i.e., a decrease of glottal incomplete closure and the increase of the amplitude of the mucosal wave and symmetry of vocal folds vibration pattern). 

One limitation of our study was the sample size. We suggest that further studies be carried out with a larger sample size and a comparative design to assess the current method against other alternatives. Furthermore, access to support technology such as the Acublade CO2 laser system may lead to an increased subtlety and precision of incisions and eventually result in a higher voice quality.

## Conclusion

The island flap procedure accompanied by a CO2 laser proved to be a safe and effective option in the treatment of intracordal mucus retention cysts. No complications of this new technique were spotted in our patients, but further investigation is required to compare this technique against other methods to further elucidate the results.
